# A Novel *cis* Element Achieves the Same Solution as an Ancestral *cis* Element During Thiamine Starvation in *Candida glabrata*

**DOI:** 10.1534/g3.119.400897

**Published:** 2019-11-15

**Authors:** Christine L. Iosue, Anthony P. Gulotta, Kathleen B. Selhorst, Alison C. Mody, Kristin M. Barbour, Meredith J. Marcotte, Lilian N. Bui, Sarah G. Leone, Emma C. Lang, Genevieve H. Hughes, Dennis D. Wykoff

**Affiliations:** Department of Biology, Villanova University, Pennsylvania 19085

**Keywords:** thiamine, *Candida glabrata*, *PDC2*, *THI2*, *cis* evolution

## Abstract

Regulatory networks often converge on very similar *cis* sequences to drive transcriptional programs due to constraints on what transcription factors are present. To determine the role of constraint loss on *cis* element evolution, we examined the recent appearance of a thiamine starvation regulated promoter in *Candida glabrata*. This species lacks the ancestral transcription factor Thi2, but still has the transcription factor Pdc2, which regulates thiamine starvation genes, allowing us to determine the effect of constraint change on a new promoter. We identified two different *cis* elements in *C. glabrata* - one present in the evolutionarily recent gene called *CgPMU3*, and the other element present in the other thiamine (THI) regulated genes. Reciprocal swaps of the *cis* elements and incorporation of the *S. cerevisiae* Thi2 transcription factor-binding site into these promoters demonstrate that the two elements are functionally different from one another. Thus, this loss of an imposed constraint on promoter function has generated a novel *cis* sequence, suggesting that loss of *trans* constraints can generate a non-convergent pathway with the same output.

The birth of genes and promoters *de novo* requires both variation and an adaptive advantage (Carvunis *et al.* 2012; Blount *et al.* 2018). There are notable examples of selection leading to intricate regulation of many genes through a signal transduction pathway, although genetic drift is frequently involved (Carroll 2008; Losos 2011; Stern 2013). Often, genes involved in a specific response appear to acquire the same *cis* sequences in their promoters and thus can be coordinately regulated by a small set of transcription factors (Tanay *et al.* 2005; Sorrells *et al.* 2018). In most cases, convergent evolution of extremely similar DNA sequences appear *de novo* through the constraints of the transcription factor only being able to bind a specific sequence (Dalal *et al.* 2016; Dalal and Johnson 2017; Kuang *et al.* 2018). Therefore, it seems likely that the same adaptive solution can evolve repeatedly if there is a selective pressure. In studying thiamine metabolism in yeast, we identified a new promoter in an existing signal transduction pathway where one of the two required transcription factors was lost, and here we observe the appearance of a novel *cis* element.

Thiamine (and its active pyrophosphorylated form – TPP) is required for critical decarboxylation reactions in the cell, and thus, is required for all life (Sriram *et al.* 2012; Osiezagha *et al.* 2013). Nosaka and colleagues have determined much of what is known about the thiamine signal transduction (THI) pathway in *S. cerevisiae* (Nosaka 2006). There are two DNA binding proteins, Thi2 and Pdc2, which interact with a regulator, Thi3. Thi3 is thought to bind TPP directly through its pyruvate decarboxylase-like domain, and when TPP is bound, the transcriptional complex is destabilized (Mojzita and Hohmann 2006; Nosaka *et al.* 2008, 2012). In low cytoplasmic thiamine conditions, TPP is not bound to Thi3, and the three-protein complex drives the high-level transcription of ∼10 genes that allow for the acquisition and/or synthesis of thiamine. A putative Thi2 binding site has been identified (which we verify here), but for Pdc2 binding, only a putative region of DNA has been identified (Nosaka 2006; Nosaka *et al.* 2012).

We previously identified that *C. glabrata*, unlike most yeast species, is auxotrophic for thiamine because of a partial loss of the biosynthetic pathway, but it still upregulates 5 genes involved in biosynthesis and scavenging (*CgTHI4*, *CgTHI20*, *CgTHI10*, *CgPET18*, and *CgPMU3*) >50 fold in response to thiamine starvation (Iosue *et al.* 2016; Nahas *et al.* 2018). Similar to *S. cerevisiae*, this upregulation is dependent on the DNA binding protein, Pdc2 and its regulator, Thi3 (Iosue *et al.* 2016; Nahas *et al.* 2018). However, *C. glabrata* lost the transcription factor Thi2, which is necessary for the thiamine starvation response in *S. cerevisiae* and in the ancestor of these yeast species, suggesting that there is some rewiring of how thiamine responsive genes are regulated (Gabaldón Estevan *et al.* 2013; Huerta-Cepas *et al.* 2014) (Figure 1).

**Figure 1:**
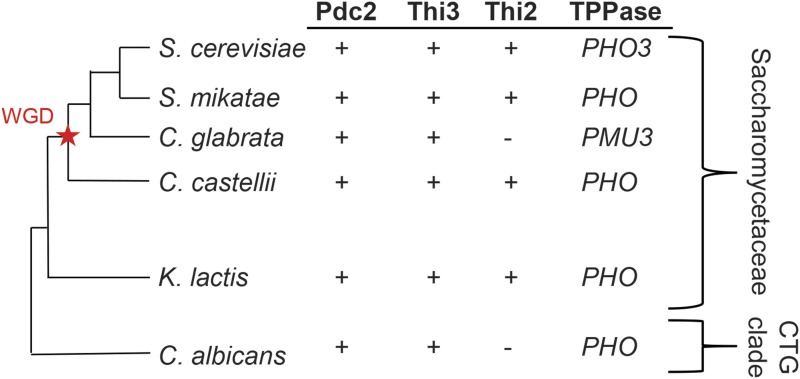
Phylogenetic relationships and presence or absence of thiamine signal transduction pathway transcription factors and thiamine pyrophosphatases (TPPases). Using a phylogeny of yeast (Gabaldón and Carreté 2016; He *et al.* 2017), presence or absence of genes was determined previously (Wapinski *et al.* 2007; Huerta-Cepas *et al.* 2014). *C. glabrata* lacks *THI2* as do the other “*glabrata* group” yeast (not presented in figure), but only *C. glabrata* contains the *PMU* array of genes (Gabaldón Estevan *et al.* 2013). We believe that the *S. cerevisiae* THI pathway behaves similar to the ancestral pathway, and *C. glabrata* has lost Thi2, gained *PMU3*, and is unable to synthesize thiamine *de novo*. WGD (and the star) refers to the whole genome duplication event, and CTG clade refers to the altered codon usage of *C. albicans*.

*C. glabrata* has also recently acquired a novel phosphatase gene (*CgPMU3*) regulated by thiamine starvation (Orkwis *et al.* 2010; Nahas *et al.* 2018). *CgPMU3* is up regulated >50-fold in response to thiamine starvation and is essential for accessing external thiamine when it is pyrophosphorylated. Interestingly, *CgPMU3* appears to have replaced the more common *PHO3*-related phosphatases observed in other related species (Nahas *et al.* 2018) (Figure 1). Because pyrophosphatase activity provides a selective advantage by allowing cells to access phosphorylated forms of external thiamine, we were able to ask the question of whether a new gene becomes integrated into a regulatory pathway in a completely novel way, or are the existing *trans* components used, but with modifications. We observed the unexpected result of a new promoter, regulated by thiamine starvation, acquiring multiple novel characteristics relative to the promoters that have been present over a long evolutionary time. However, the promoter behaves very similar to the ancestral promoters in terms of output and uses some of the same ancestral *trans* factors. Thus, the experiment of “replaying life’s tape” (Gould 1990; Blount *et al.* 2018) by looking at a new promoter under selection suggests parallel yet very different changes and interestingly, the generation of a new DNA binding element.

In addition to observing a novel *cis* element, this work is motivated by defining the requirements for the *CgPMU3* promoter. *C. glabrata* resides predominantly in mammalian gastrointestinal tracts, and is the second most common cause of candidiasis (Gabaldón *et al.* 2016; Pappas *et al.* 2018; Kumar *et al.* 2019). *C. glabrata* is often more resistant to anti-fungal drugs relative to *C. albicans* and thus the development of targeted therapies would be beneficial (Whaley and Rogers 2016). Because human serum transports thiamine primarily in the form of TPP, an understanding of *CgPMU3* upregulation is critical for long-term studies about the pathogenicity of this species (Lu and Frank 2008), and targeting *CgPMU3* expression is a potential avenue for antifungal development.

Here, we used truncation analysis to identify regions of THI promoters required for upregulation of expression during thiamine starvation. We identified an 11 base pair (bp) region that is essential for upregulation in *CgPMU3* but surprisingly, regions similar to this in other THI promoters were not required for upregulation. Using almost base pair resolution, we identified a different 13 bp region in other THI promoters that does not share obvious similarity to the 11 bp region in *CgPMU3*, and these 11 bp and 13 bp regions are not interchangeable. The difference between *CgPMU3* and other THI promoters is that *CgPMU3* likely never evolved thiamine regulation in the presence of Thi2, unlike the other THI promoters. We examined expression of *C. glabrata* THI promoters in *S. cerevisiae*, and noted they are not regulated, but the inclusion of a putative Thi2 binding site restored upregulation of *Cg*THI promoters (and *CgPMU3*) in *S. cerevisiae*. However, this upregulation differed between the ancestral THI promoters and the *CgPMU3* promoter. We conclude that loss of Thi2 and selection for a newly regulated gene confers a different path across the evolutionary landscape than the THI genes that are conserved within the *Ascomycete* lineage.

## Materials and Methods

### Strains

Most of the experiments were performed in *C. glabrata* wild-type (Cormack and Falkow 1999) and *S. cerevisiae* wild-type (Wykoff and O’Shea 2001) strains. Additional strains used in this study were deletions of the thiamine pathway regulators: *Cgthi3*∆ (DG141), and *Cgpdc2*∆ (DG271), *Scthi2*∆ (DC126), *Scthi3*∆ (DC143) (Iosue *et al.* 2016) and *Scpdc2*∆, which was generated in this this study. Because *PDC2* is essential in glucose-containing medium in *S. cerevisiae*, *NATMX6* was amplified using PCR (primers in Supplemental Material, Table S1) and transformed into a diploid strain to delete *ScPDC2*. We covered this deletion with a URA3^*+*^ plasmid (pRS316) containing *ScPDC2*. Through random sporulation, we identified haploid colonies that were *Scpdc2*Δ. To construct a *Scpdc2*∆ strain capable of growth in glucose medium, *ScPDC1* was overexpressed in this strain: *ScPDC1* was amplified by PCR and cloned by homologous recombination (Corrigan *et al.* 2013) into a *pdc2*∆ strain on a LEU2*+* plasmid (pRS315) under the control of the *ScADH1* promoter. This strain was then grown on SD (synthetic dextrose, Sunrise Science, CA) plates with 5-FOA to select against the URA3^*+*^ plasmid containing *ScPDC2*. For sequencing of the *CgPMU3* promoter in the SEL-seq experiment, the entire *PMU* gene family (*PMU1*, *PMU2*, and *PMU3* promoter and open reading frame) was deleted with *NATMX6* in a *C. glabrata* wild-type strain (Table S1).

### Plasmid Construction

To assay induction of THI pathway genes, we constructed plasmids where either the full-length promoters (1000 bp) or smaller regions of the promoters of these genes were driving expression of yellow fluorescent protein (YFP). The promoters were amplified by PCR (Table S1) and cloned by homologous recombination into a HIS3*+* plasmid (pRS313) containing YFP in a wild-type strain (Corrigan *et al.* 2013). To investigate the effects of mutations/deletions in the promoters, PCR was used to amplify the full-length promoter in two regions, with overlapping primers that incorporated the altered sequence (Table S1), and these PCR products were cloned into a YFP plasmid as previously described (Corrigan *et al.* 2013; Nahas *et al.* 2018). For some promoters, a *Pac*I restriction enzyme site replaced the UAS so the opposite UAS could be easily introduced. Details of cloning are available upon request.

### Flow cytometry

To measure induction of the THI pathway genes, fluorescence of cells containing plasmids with promoters driving YFP was quantified by flow cytometry. Cells were grown at 30° in thiamine replete SD medium lacking histidine (Sunrise Science, CA) to logarithmic growth phase (OD_600_ 0.2-0.5). Cells were harvested by centrifugation, washed 3 times with sterile water, inoculated into thiamine replete (0.4 mg/L) and starvation (no thiamine added) conditions in SD medium lacking histidine, and grown at 30° overnight (∼18 h). Mean fluorescence (in arbitrary units, a.u.) of each strain was measured using a fiow cytometer with a 533/30 FL1 filter set (Accuri C6, BD Biosciences). In almost all cases, background fluorescence was less than 12,000 a.u.; however, there is variability of fluorescence based on precise growth conditions and we included positive and negative controls in each experiment.

### SEL-seq sequencing

To perform the SEL-seq experiment with the *CgPMU3* 11 bp element, we constructed a plasmid that contained the *ScTHI5* ORF in frame with YFP and the *CgPMU3* promoter. To make the promoter, we used PCR to generate a ∼250 bp product that incorporated Ns in the 11 nucleotide region with ∼30 bp of homology to a ∼750 bp PCR product corresponding to the rest of the *CgPMU3* promoter (from -1000 bp to -250 bp). The three PCR products – two *CgPMU3* promoter PCR products and the *ScTHI5* open reading frame (Table S1) – were gap repaired (Corrigan *et al.* 2013) into a strain lacking the wild-type *CgPMU3* promoter (*Cgpmu1-3∆NATMX6* described above) and we collected 131,000 independent transformants. Approximately, 5% of transformants were judged as highly expressing during thiamine starvation (based on YFP expression). We pooled the transformants and took a time zero sample for deep sequencing of the *CgPMU3* promoter. Based on sampling of unique sequences, we generated ∼90,000 unique sequences to query. We then grew the cultures in SD medium lacking thiamine and histidine for three successive days with 1:1000 dilution every 24 h (allowing ∼20 generations to pass). We monitored fluorescence by flow cytometry and observed the frequency of cells that were highly fluorescent jump from 6 to >90% in 24 h. We collected three independently grown cultures (in medium lacking thiamine) to purify DNA and amplify the *CgPMU3* promoter for next generation sequencing on a MiSeq (Illumina, San Diego, CA). Sequences were extracted in Geneious, and at least 2x1750 sequences were analyzed for each sample. We sorted the sequences, identified the number of unique sequences, and quantified the percent representation of the sequence in the total sequences. We verified that extraction of a different subset did not alter the results – *i.e.*, the same sequences were repeatedly identified as enriched.

### Data availability

All strains, plasmids, and raw data are available upon request. Table S1 lists the primers used in this study to generate strains and plasmids. Figure S1 demonstrates that *ScTHI5* confers a growth advantage to *C. glabrata* during thiamine starvation. Figure S2 shows the frequency of abundant sequences after selection in thiamine starvation in the SEL-seq experiment. Table S2 shows the raw data from the seven samples sequenced in the SEL-seq experiment. Table S3 lists the sequences that were highly enriched after selection in thiamine starvation. Figure S3 aligns the sequences in Table S3 with *C. glabrata* THI promoters. Figure S4 shows a scanning mutagenesis of the 13 bp THI UAS in the *CgPET18* promoter. Figure S5 is a schematic of *S. cerevisiae* promoters with the locations of binding sites as well as mutations and deletions made in this study. Figure S6 demonstrates that *S. cerevisiae* THI promoters are dependent on Pdc2, Thi2, and Thi3. Supplemental material available at figshare: https://doi.org/10.25387/g3.10308194.

## Results

### The CgPMU3 promoter contains an 11 bp element required for thiamine starvation upregulation

**Figure 2:**
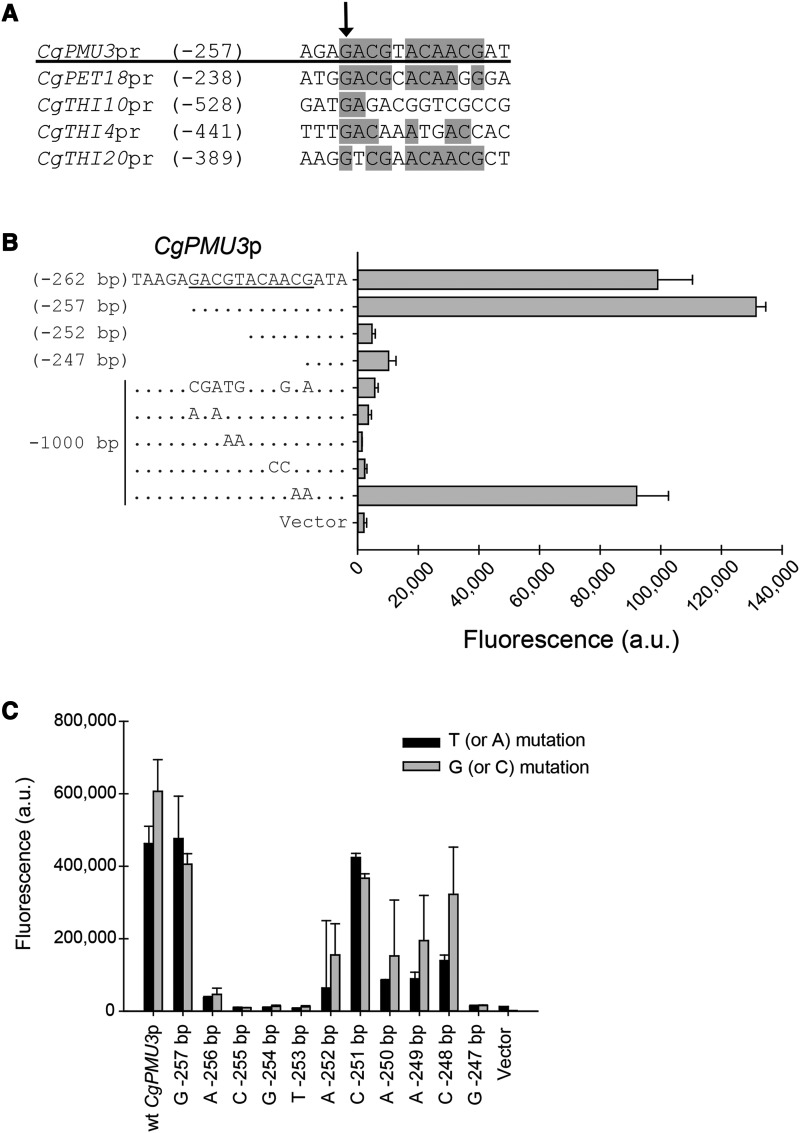
The *CgPMU3* promoter contains an 11 bp UAS necessary for thiamine starvation dependent expression. A) After truncation analysis of the *CgPMU3* promoter, a MEME analysis identified a region that appeared conserved in THI promoters (1000 bp of each THI promoter and 270 bp of the *CgPMU3* promoter). Searching the *C. glabrata* genome for a consensus GACRNANNACG using a pattern match algorithm (Skrzypek *et al.* 2017), yielded 116 genes with this element in the 1 kb upstream of the start codon, including *CgPMU3*, but no other known THI regulated genes. The gray shading indicates nucleotides in common with *CgPMU3*. The number after the promoter name indicates the nucleotide (under the arrow) upstream from the start codon. B) Characterization of the 11 bp *CgPMU3* UAS. The first four samples show truncation analysis and the next five samples have mutations introduced into the full-length (1000 bp) wild-type promoter. Promoter induction was assayed during thiamine starvation by measuring the fluorescence of cells containing plasmids with these promoters driving YFP. C) Scanning mutagenesis of the 11 bp *CgPMU3* UAS. Single mutations were introduced into the full-length promoter, replacing the native nucleotide with either a T or a G, except when the native nucleotide was a T/G, in which case the T/G was replaced with an A/C. For this and the following figures, the data presented is the mean and standard deviation of at least three independently grown samples.

To understand the DNA sequences required for upregulation by thiamine starvation, we undertook promoter truncation experiments with portions of the *CgPMU3* promoter fused to the open reading frame of yellow fluorescent protein (YFP). Induction of the promoter was quantified using flow cytometry to measure the fluorescence of YFP in the cells. First, we truncated in 100 bp increments and then in 20 bp increments from -1000 bp (referring to the location upstream of the start codon) to the start codon (data not shown). We narrowed the beginning of the upstream activating sequence (UAS) to between -260 bp and -240 bp. Performing a MEME motif discovery analysis (Bailey *et al.* 2009), we identified an 11 bp region that appeared to be somewhat conserved in other THI promoters (Figure 2A). To determine whether this region was important for upregulation, we further truncated the *CgPMU3* promoter and made point mutations in the 11 bp region in the context of the full-length 1000 bp promoter (Figure 2B). These data indicate that numerous nucleotides in the 5′ GACGTACAACG 3′ sequence are critical for high-level de-repression of the *CgPMU3* promoter.

To identify the importance of each nucleotide in the 11 bp sequence, we mutated individual nucleotides in the context of the 1000 bp promoter to either a T or a G (Figure 2C). When a T or G was present in the original sequence, we mutated it to either an A or C, respectively. The trends between the two scanning mutagenesis experiments were similar, and they identified the bases in uppercase as being critically important for thiamine regulation – gACGTacaacG. However, it is clear that other nucleotides have importance, as mutation of two As (that are next to one another) to Cs also disrupts the function (Figure 2B). While there is variable conservation of these nucleotides in other THI promoters based on the MEME analysis, using only the essential nucleotides to search the *C. glabrata* genome identifies too many sequences to be informative.

### The 11 bp UAS in CgPMU3 is not important for other THI promoters

**Figure 3:**
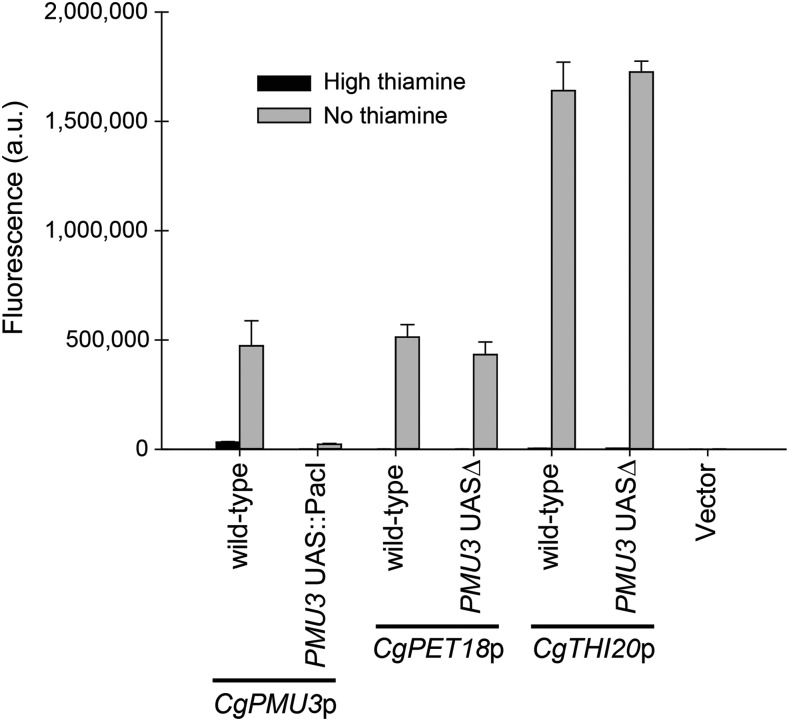
Deletion of the 11 bp *CgPMU3* UAS in THI promoters does not eliminate thiamine starvation dependent expression. The putative *CgPMU3* UAS (Figure 2A) was precisely deleted in the full-length promoters of *CgPMU3* (and replaced with a *Pac*I restriction site), *CgPET18*, and *CgTHI10* and assayed for YFP expression in high and no thiamine conditions. While necessary for *CgPMU3*, this UAS is not important for induction of other THI promoters.

To determine whether the element identified in the *CgPMU3* promoter was important for upregulation in other THI promoters, we deleted the 11 bp element in *CgPET18* and *CgTHI20*. We chose these two promoters because they shared the most sequence similarity to the *CgPMU3* UAS (with 9 nucleotides identical out of 11, Figure 2A). We did not find a major defect in transcriptional induction of these genes when the putative *PMU3* UAS was deleted (Figure 3). This was remarkable, given the common sequence with the *CgPMU3* element. However, neither *CgPET18* nor *CgTHI20* have the strict xACGTx_5_G motif. We hypothesize that we have identified this motif for one of two reasons. Either 1) this motif has appeared by chance in the promoters, as the sequences are imperfect matches with the *CgPMU3* UAS, or 2) this motif is present, but does not have a critical role in thiamine starvation regulation by our assay. Regardless, this suggests that *CgPMU3* appears to have a different UAS requirement from the other THI genes. It is worth noting that *CgPMU3* is a recent duplicate of a phosphatase gene and only acquired thiamine regulation in *C. glabrata* (Gabaldón *et al.* 2016; Nahas *et al.* 2018), whereas the other THI genes are present in multiple *Ascomycota* species and have likely been regulated by the same THI pathway through multiple speciation events.

### SEL-seq approach to identifying the critical nucleotides in the CgPMU3 promoter

Because the *CgPMU3* UAS did not appear important in other THI promoters, we wanted to take a relatively unbiased approach to understand what nucleotides were important for upregulation in *CgPMU3* and to determine if there were sequences that conferred regulation that might have similarity to *cis* elements in other THI promoters. We hypothesized that multiple versions of the *CgPMU3* UAS would confer thiamine starvation regulation in this 11 bp element – *i.e.*, there is some degeneracy in the sequence, and that potentially alterations in the *CgPMU3* UAS might cause it to resemble elements in other THI promoters. To identify the important nucleotides in the 11 bp UAS using an unbiased approach, we performed a modified SEL-seq experiment (Farley *et al.* 2015). Using a selection with theoretically 4.2 million (4^11^) possibilities being queried for high-level expression during thiamine starvation, we replaced the 11 bp UAS with all four nucleotides in each position (incorporated into a primer – Table S1) and selected for high-level expression during thiamine starvation. Because *C. glabrata* is auxotrophic for thiamine and addition of *ScTHI5* restores prototrophy (Iosue *et al.* 2016), we could select for high-level expression of the *CgPMU3* promoter by having it control *ScTHI5* transcription during thiamine starvation. We confirmed that this *CgPMU3*p-*ScTHI5* plasmid was capable of supporting growth of *C. glabrata* in the absence of thiamine (Figure S1).

Using a fusion PCR method, we gap repaired the *CgPMU3* promoter upstream of the *ScTHI5* ORF, replacing the *CgPMU3* UAS with all four nucleotides, allowing multiple sequence options to replace the UAS. The selection was successful but limited in terms of exploring the 4 million possibilities. We obtained 10^5^ transformants, and observed ∼90,000 unique sequences with the 20 most abundant sequences representing 9.7% in our sampling sequencing prior to selection (Figure S2 and Table S2). It is likely that PCR and primer synthesis introduced biases that led to a few abundant sequences, and a diversity of other sequences. However, after selection, the 20 most abundant sequences (which were different from the preselection sequences) represented on average 72.5% of the total sequences, indicating that some sequences conferred a strong selective advantage. To begin to eliminate sequences that might simply confer a high level expression independent of the THI pathway, we performed a parallel selection in a *Cgpdc2*Δ strain, expecting that if a sequence was abundant in the *Cgpdc2*Δ strain, that it was a sequence that allowed for higher-level expression of the *ScTHI5* construct independent of the THI pathway (Figure S2). Eight sequences were >90 fold enriched in a THI pathway dependent manner, and all contained a 5′-CTG-3′ motif (Table S3). Interestingly, two sequences that we identified were a 10/11 bp and 8/11 bp match for a sequence element in the *CgPET18* promoter, different from the *PMU3* UAS in Figure 2A, indicating that the other THI promoters contain a sequence that might functionally replace the *CgPMU3* UAS (sequences A and G in Figure S3).

### A 13 bp THI UAS in non-CgPMU3 THI promoters is important for thiamine starvation regulation

**Figure 4:**
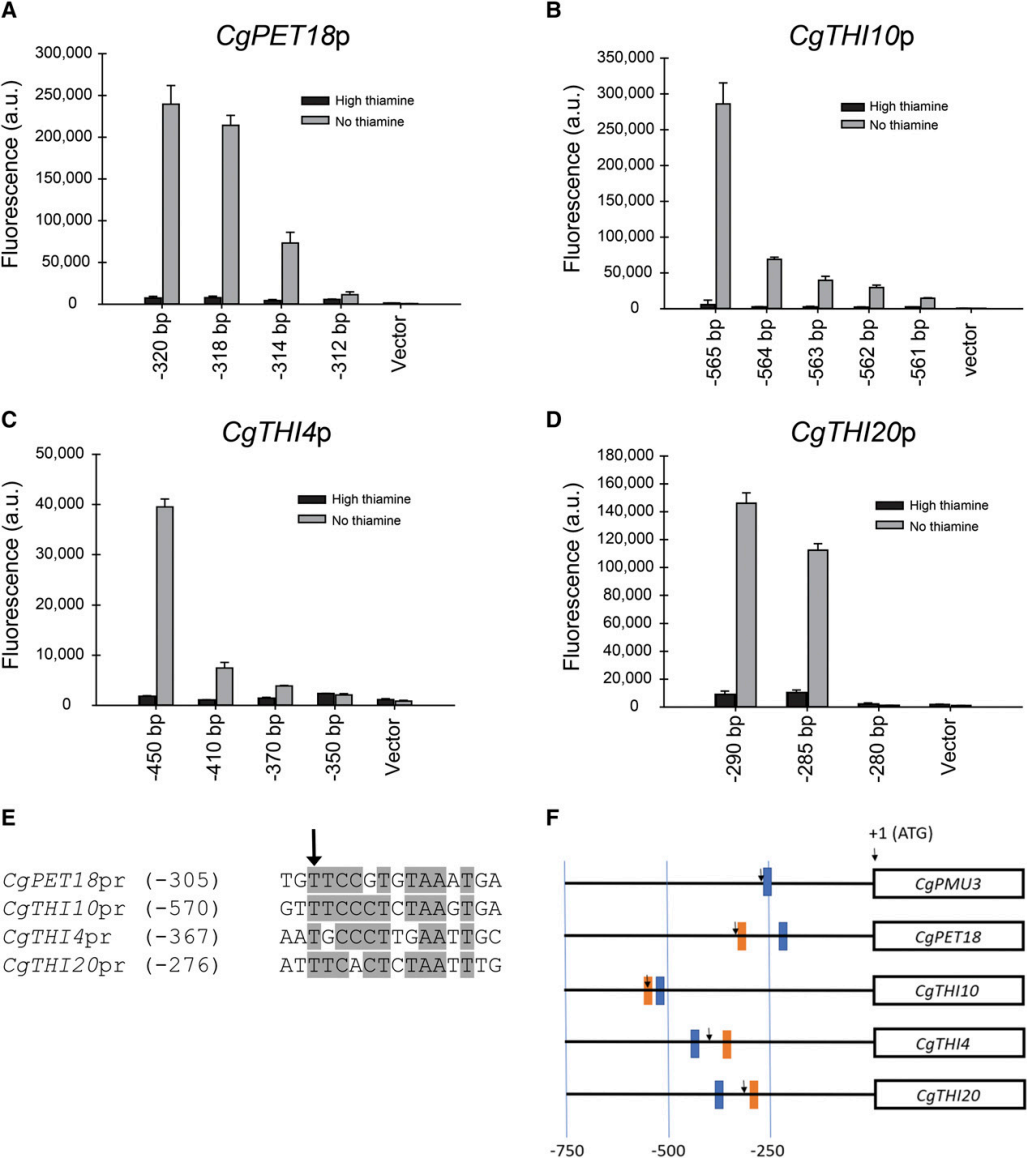
Fine scale truncation analysis of THI promoters uncovers a 13 bp UAS that is not present in *CgPMU3* A-D) We truncated THI promoters in 100 bp intervals and then further narrowed down to regions where we observed a >90% decrease in thiamine starvation induction. E) With 50 bp regions of the THI promoters around the site of truncation, we performed a MEME analysis and identified a 13 bp region which was not present in the 1000 bp *CgPMU3* promoter. The TTCCCTBTAAWTG consensus is only found in 4 promoters in the *C. glabrata* genome, and those genes do not appear to be regulated by thiamine starvation based on previous RNA-seq data (Nahas *et al.* 2018). Each promoter element has at least one mismatch from the consensus, suggesting some permissiveness in the element. The arrow indicates the nucleotide number upstream from the start codon and the gray shaded regions are conserved nucleotides. F) A schematic of the location of the two elements in the five most upregulated THI pathway promoters with the arrows indicating where a truncation reduced expression. The blue boxes correspond to the *CgPMU3* UAS and the orange boxes correspond to the THI UAS.

To determine regions that are important for thiamine regulation in THI promoters other than *CgPMU3*, we took a parallel approach to the *CgPMU3* promoter, and truncated the *CgPET18*, *CgTHI10*, *CgTHI4*, and *CgTHI20* promoters. We narrowed down the beginning of a regulatory sequence in these promoters to a few base pairs (Figure 4A-D). After a MEME analysis, we identified a new DNA sequence that does not appear to be present in the *CgPMU3* promoter (Figure 4E), that closely correlates with the locations of where truncation begins to decrease thiamine starvation regulation (arrows in Figure 4F), and that overlaps with the SEL-seq *CgPET18* sequence (Figure S3). Only the *CgPET18* promoter sequence contains a 5′-CGT-3′ motif that is critical for the *CgPMU3* UAS element, perhaps explaining why we enriched for *CgPET18* elements in the SEL-seq experiment.

**Figure 5:**
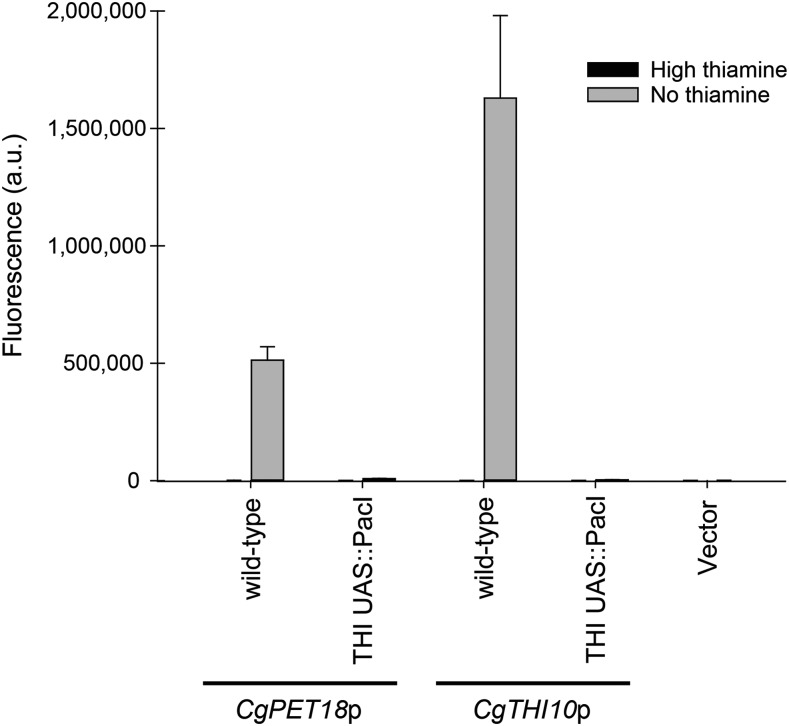
Deletion of the THI UAS eliminates thiamine starvation inducible expression of THI promoters. The putative THI UAS (Figure 4E) was precisely deleted, and replaced with a *Pac*I restriction enzyme site, in the full-length promoters of *CgPET18* and *CgTHI10* and assayed for YFP expression in high and no thiamine conditions.

To validate that these newly identified conserved sequences were important for thiamine starvation regulation, we replaced the 13 bp UAS with a *Pac*I restriction enzyme site in the context of the 1000 bp promoter and determined whether this element is critical for upregulation of the *CgPET18* and *CgTHI10* promoters (Figure 5). In both promoters, deleting the UAS decreased expression during thiamine starvation. We also performed a scanning mutagenesis of the *CgPET18* full-length promoter, mutating these 13 bp individually to A (or C if an A was in that position), and determined that the xxCCGTxxAxxTG nucleotides were important for expression (Figure S4). There is overlap between the *CgPMU3* and THI UAS in terms of both possessing a 5′-CGT-3′; however, CGT is not absolutely required as *CgTHI10* does not contain this sequence, and the remaining nucleotides are not easily aligned with the *CgPMU3* UAS. Thus, we have identified two UAS elements that do not appear related to one another: the *CgPMU3* UAS (Figure 2) and the THI UAS present in all of the other THI promoters (Figure 4).

**Figure 6:**
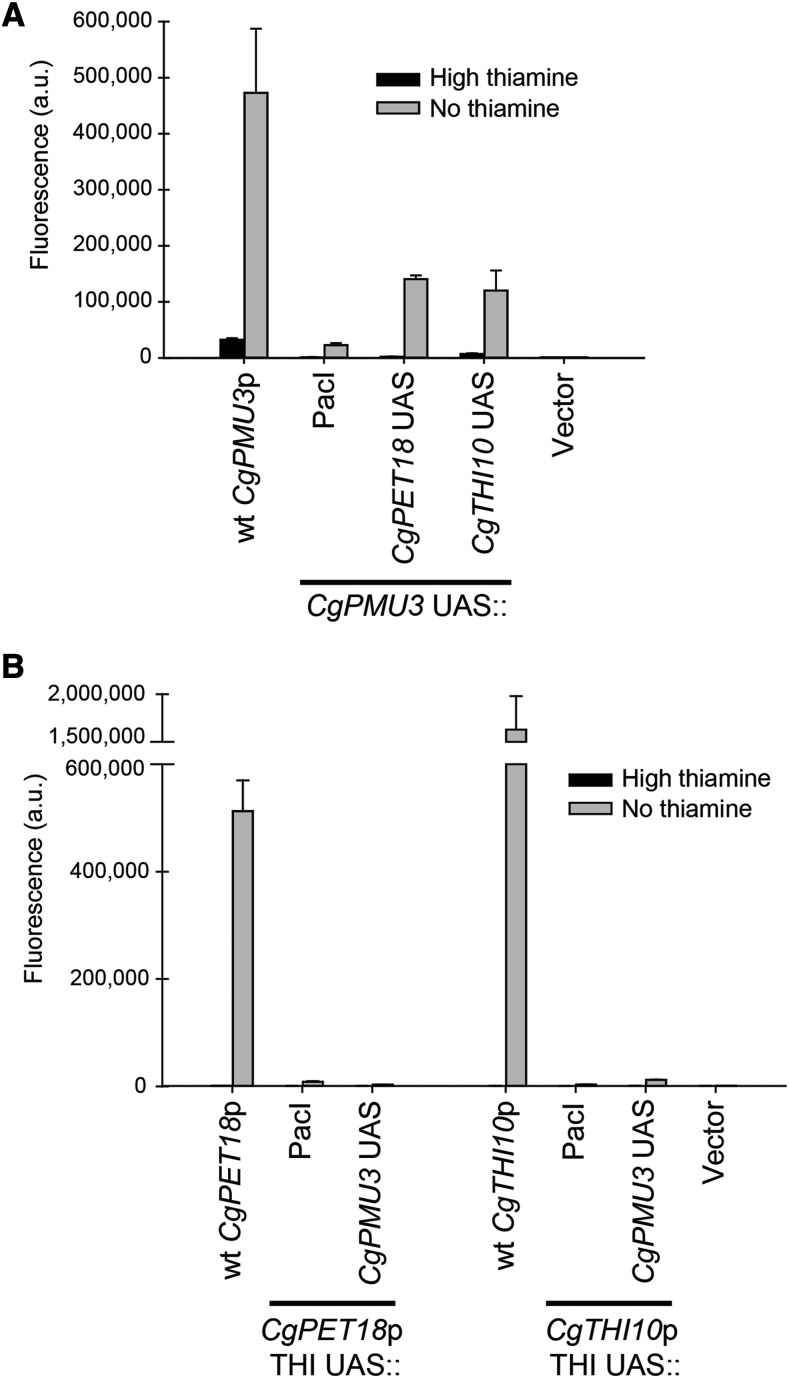
The THI UAS is able to substitute for the *CgPMU3* UAS, but the *CgPMU3* UAS cannot replace the THI UAS. A) Deletion of the *CgPMU3* UAS in the context of the full-length *CgPMU3* promoter results in a severe defect in thiamine starvation inducible expression of YFP; however, replacement of the *CgPMU3* UAS with either the *CgPET18* or *CgTHI10* UAS restores upregulation of the *CgPMU3* promoter. B) Deletion of the THI UAS in the context of the full-length promoter results in a severe defect in thiamine starvation inducible expression of YFP; however, replacement of the *CgPET18* UAS or the *CgTHI10* UAS with the *CgPMU3* UAS does not restore upregulation of the promoters.

We next determined whether the *CgPMU3* UAS or the THI UAS were capable of substituting for one another in promoters. To do this, we deleted the critical element with a *PacI* restriction enzyme site and used this *PacI* site to incorporate the opposite element (Figure 6). The THI UAS is partially capable of substituting for the *CgPMU3* UAS (Figure 6A), which is not surprising, given the SEL-seq data where a randomly selected sequence in the *CgPMU3* promoter is very similar to the *CgPET18* 13 bp UAS. However, the *CgPMU3* UAS is not capable of replacing the THI UAS (Figure 6B), suggesting that this recently evolved 11 bp promoter element does not function identically to the 13 bp element.

### The THI UAS is similar to sequences in S. cerevisiae promoters and is likely the ancestral UAS

**Figure 7:**
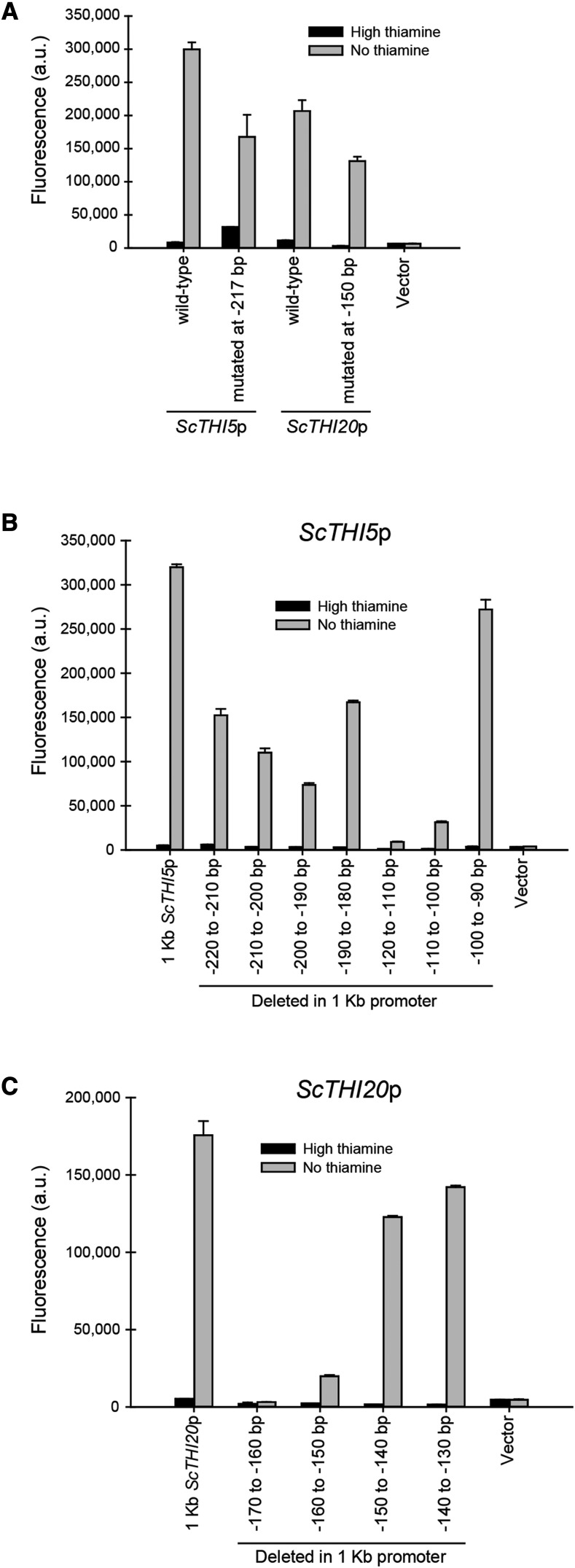
Deletion of regions most similar to the THI UAS in *S. cerevisiae* promoters does not abrogate thiamine starvation regulation, but deletion of regions near the UAS reduces expression. A) Mutation of the region most similar to the THI UAS has little effect on upregulation in two *S. cerevisiae* promoters (see Figure S5 for details on the sequence). B) A scanning deletion of the promoter region of *ScTHI5* and C) *ScTHI20* uncovers 20 bp that appear important for expression. These regions span the Thi2 binding site and are near the putative *Cg*THI UAS.

Because *PET18*, *THI10* (*TH17*), *THI4*, and *THI20* are present in the genomes of *Saccharomycetaceae* and regulated by thiamine starvation, we consider these genes and promoters to have been present in the common ancestor (Byrne and Wolfe 2005; Gabaldón Estevan *et al.* 2013; Huerta-Cepas *et al.* 2014). Conversely, *CgPMU3* is a novel gene present only in *C. glabrata* (Orkwis *et al.* 2010; Nahas *et al.* 2018). To determine whether the THI UAS in *C. glabrata* is similar to a UAS in *S. cerevisiae*, we identified through MEME-suite analysis the regions in *S. cerevisiae* that are most similar to the 13 bp UAS (Figure S5). We then mutated them in the *ScTHI5* and *ScTHI20* promoters, and assayed the ability of these promoters to induce expression during thiamine starvation (Figure 7A). Surprisingly, deletion of this element did not disrupt expression of these genes during thiamine starvation. However, deletion of regions near this site (within 20 bp and spanning the putative Thi2 binding site: at -110 bp in *ScTHI5*p and at -170 bp in *ScTHI20*p) did disrupt upregulation (Figure 7B and 7C and Figure S5). Using computational methods, there is a low confidence sequence of 5′-tatatgta-3′ as a Pdc2 binding site (Reddy *et al.* 2007; de Boer and Hughes 2012), but if there is degeneracy or error, this site could be in many locations, as we note in Figure S5. A detailed dissection of the *S. cerevisiae* promoters is warranted and this is in process in our laboratory. Ultimately, we were surprised that the 13 bp THI UAS was not required for expression, but given our results later with the incorporation of a Thi2 binding site into *C. glabrata* THI promoters, we believe that the *Sc*Pdc2 binding site may be highly degenerate, or not even required in all contexts.

### Thi2 dependence - CgPMU3 UAS with *Sc*Thi2 is different from the THI UAS with *Sc*Thi2

**Figure 8:**
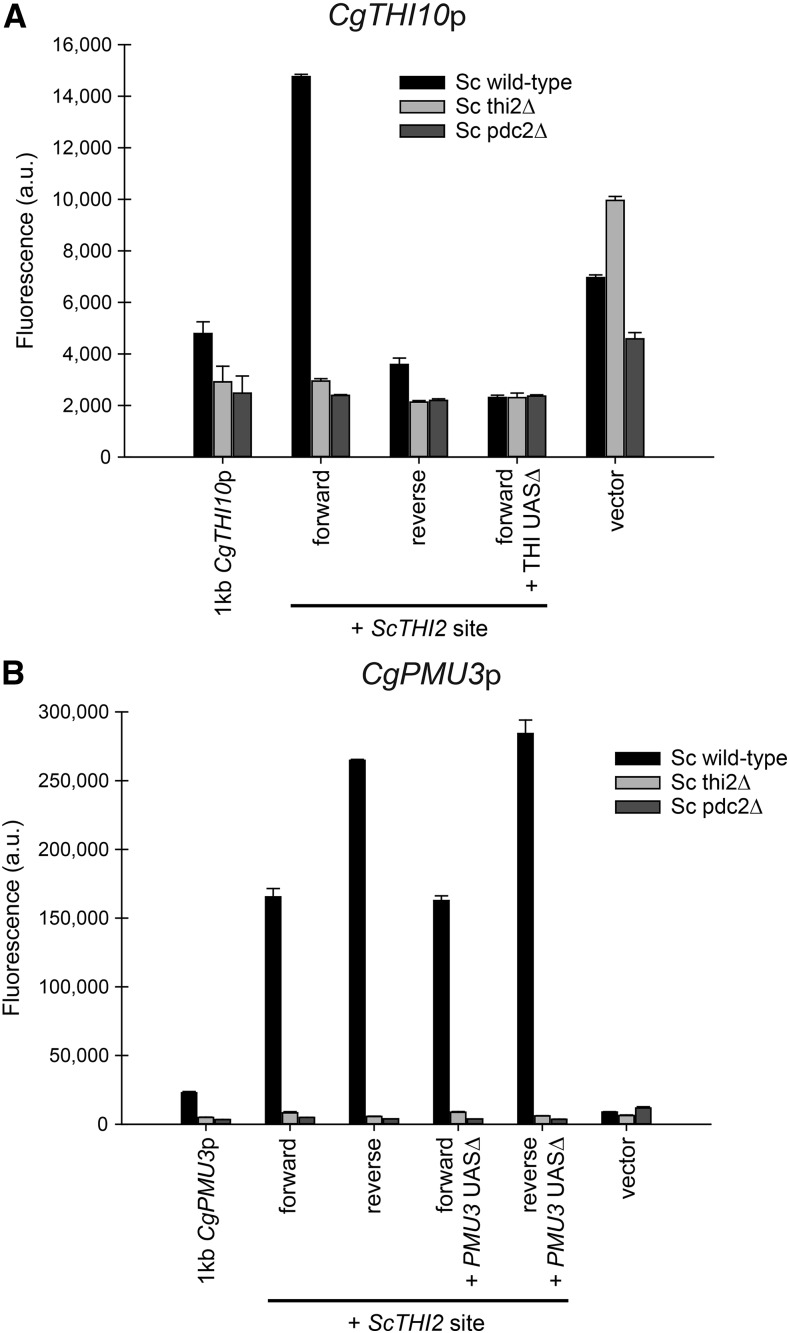
*CgTHI10* and *CgPMU3* respond differently to the introduction of a *Sc*Thi2 binding site. A) A *Sc*Thi2 binding site (forward and reverse orientation) was introduced into the *CgTHI10* promoter with and without the THI UAS deleted. These plasmids were transformed into *S. cerevisiae* strains and assayed for fluorescence in thiamine starvation conditions. For there to be increased expression of *CgTHI10* in *S. cerevisiae*, the Thi2 binding site must be incorporated in the forward orientation and expression requires the THI UAS. B) The *CgPMU3* promoter tolerates the *Sc*Thi2 binding site in either orientation and does not require the *CgPMU3* UAS to function in *S. cerevisiae*, but expression is still Thi2 and Pdc2 dependent. *CgPMU3* with a Thi2 binding site leads to higher level expression of the promoter in *S. cerevisiae* relative to *CgTHI10*. It is unclear why the two promoters have such different expression levels.

The data in Figure 6 with the switching of the UAS elements suggest that the *CgPMU3* promoter fundamentally behaves differently from the other THI promoters, but the THI and *CgPMU3* promoters are still dependent on the two known transcriptional regulators *Cg*Pdc2 and *Cg*Thi3 (Iosue *et al.* 2016). Because *CgPMU3* evolved recently, and likely did not experience selective pressures from the ancestral Thi2, we hypothesized that it may behave differently in a setting where Thi2 is important. To test this, we cloned a putative *Sc*Thi2 binding site from *ScTHI20* (Nosaka 2006) into the *CgTHI10* and *CgPMU3* promoters 5 bp upstream of the THI or *CgPMU3* UAS. We chose the *ScTHI20* Thi2 binding site because it is highly conserved in *ScTHI20* promoters across the *Saccharomyces* genus (Kellis *et al.* 2003). We were unsure which orientation would work, as the site appears to be in either orientation in other promoters (Nosaka 2006), therefore we cloned the Thi2 binding site in both the 5′ggaaacccttagag 3′ “forward” orientation and the 5′ ctctaagggtttcc 3′ “reverse” orientation. We had already determined that none of the *C. glabrata* THI promoters (including *CgPMU3*) were regulated in *S. cerevisiae* (Figure 8, and data not shown), and we asked whether the inclusion of a *Sc*Thi2 binding site altered the ability of the *C. glabrata* promoter to be regulated in *S. cerevisiae*.

Inclusion of the *Sc*Thi2 binding site allowed for regulated expression in *S. cerevisiae* for both the *CgTHI10* and the *CgPMU3* promoters (Figure 8). However, the two promoters’ dependence on the transcription factors is altered. The *CgTHI10* promoter is *ScTHI2* and *ScPDC2* dependent and requires the 13 bp UAS in concert with the *Sc*Thi2 binding site (Figure 8A). However, *CgTHI10* cannot tolerate the *Sc*Thi2 binding site in a reverse orientation, suggesting that there is an important quaternary interaction between Thi2 and Pdc2 to position the RNA polymerase machinery. In many ways, the introduction of the *Sc*Thi2 binding site has converted the *CgTHI10* promoter into a standard *S. cerevisiae* THI promoter, albeit not nearly as efficient, as the amount of expression is only double the background level of fluorescence.

In contrast to *CgTHI10*, *CgPMU3* has acquired upregulation in a different manner. First, the inclusion of the *Sc*Thi2 binding site can be in either orientation to confer upregulation, although there appears to be a preference for the reverse orientation for maximal expression (Figure 8B). Second, while the *CgPMU3* promoter in *S. cerevisiae* requires both *ScTHI2* and *ScPDC2*, it is unclear where *Sc*Pdc2 binds, as loss of the 11 bp UAS has no effect on the upregulation. The *CgPMU3* promoter has not adopted a behavior like other THI promoters, but appears to be regulated because *Sc*Thi2 is able to bind to the promoter, and likely *Sc*Pdc2 has accompanied *Sc*Thi2 because it is in a complex with it, and *Sc*Pdc2 allows for the recruitment of the RNA polymerase machinery. Thus, we conclude the *CgPMU3* promoter has a significantly different *cis* architecture from other THI promoters, and this is likely a consequence of the lack of co-evolution with Thi2.

## Discussion

We have identified two unrelated UASs in thiamine starvation-regulated promoters in *C. glabrata*. One UAS is likely similar to the common ancestor of THI promoters, where the transcription factor Thi2 was present during the selection for thiamine regulation. This THI UAS is likely a relatively degenerate sequence that is able to recruit Pdc2. The *CgPMU3* UAS is new and likely never experienced selection with Thi2 present. We have determined that the UASs are not interchangeable for one another and that they function differently from one another based on how they behave with a *Sc*Thi2 binding site introduced.

**Figure 9:**
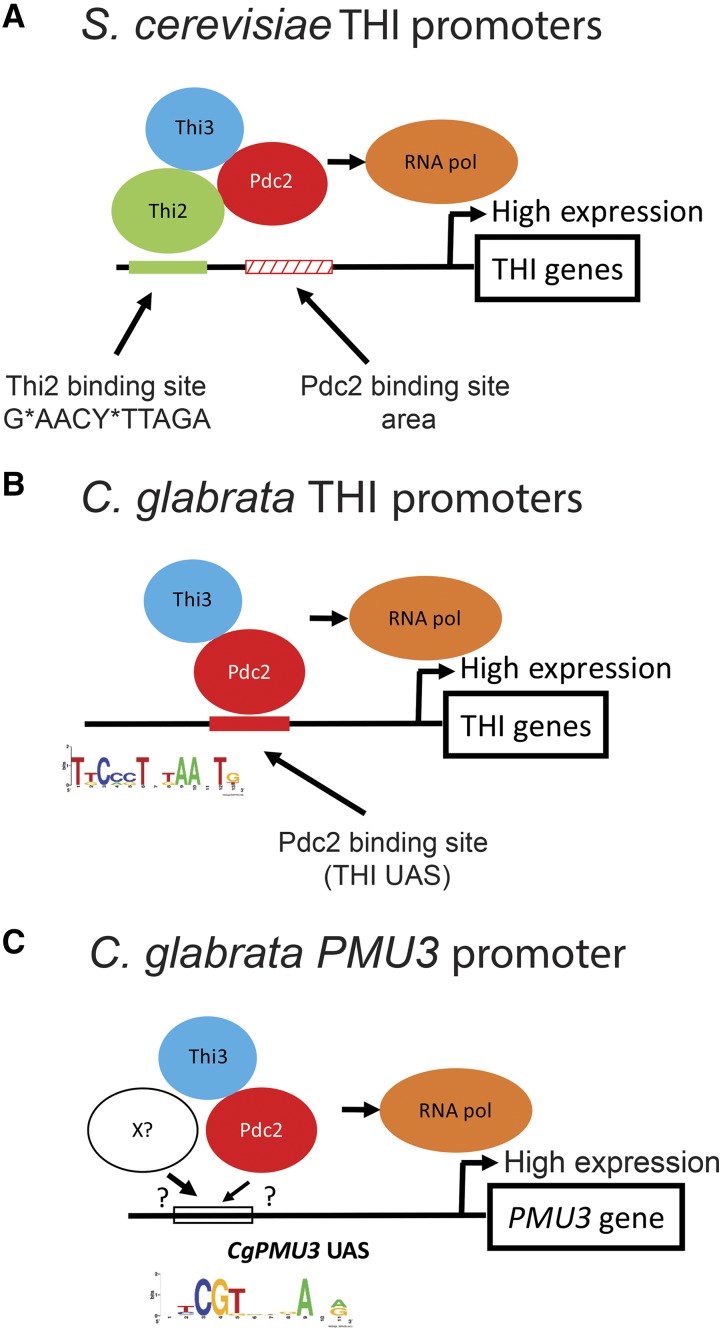
Model of transcription factor binding sites in thiamine starvation regulated promoters in *S. cerevisiae* and *C. glabrata*. A) *Sc*Thi2 binding may be the “anchoring” step, and because Pdc2 is in a complex with Thi2, Pdc2 is then able to bind to degenerate sequences nearby, leading to the recruitment of the transcriptional machinery. B) THI promoters in *C. glabrata* (other than *CgPMU3*) behave similar to *S. cerevisiae* promoters but only require Pdc2 and Thi3. C) For the *CgPMU3* promoter, it seems likely that a novel transcription factor has been co-opted into the THI pathway to act as a functional analog to Thi2, and it may bind both the *CgPMU3* UAS and *Cg*Pdc2. Regardless of where Pdc2 binds, it is still required for the recruitment of the transcriptional machinery.

While more work is required to understand how the architectures of these two promoters work, we hypothesize that *S. cerevisiae* THI promoters behave as presented previously. That is, when the intracellular TPP concentration is low, Thi2 and Pdc2 bind with Thi3 to drive transcription (Figure 9A). However, our work suggests that Thi2 binding is the “anchoring” step, and because Pdc2 is in a complex with Thi2, Pdc2 is then able to bind to degenerate sequences nearby, leading to the recruitment of the transcriptional machinery. This alteration in the model is supported by strong conservation of a Thi2 binding site in *S. cerevisiae* THI promoters, but a weak conservation of the THI UAS that we identified in this study (Kellis *et al.* 2003). Pdc2 is still required for transcription, but there is not a clear site for its binding. The inability to gel shift Pdc2 to THI DNA elements through EMSA experiments, and the very weak interaction of the DNA binding domain of *Sc*Pdc2 with a single DNA element that is Thi2 independent, suggest there is not a high affinity DNA-transcription factor interaction (Nosaka *et al.* 2012). Additionally, while deletion of *THI2* removes the majority of expression of THI promoters, there is still some induction in the absence of Thi2 (Figure S6), and overexpression of *THI3* can compensate for the loss of *THI2* presumably by making Pdc2 fully active while in a complex with Thi3 (Iosue *et al.* 2016). However, loss of *PDC2* removes all induction in response to thiamine starvation, suggesting Pdc2 is core to the transcriptional response. Thi2 may be an important anchoring transcription factor in *S. cerevisiae* THI promoters that facilitates Pdc2 transcription factor binding. Thus, Thi2 appears to be both a specificity and high-level expression factor for THI genes, and Pdc2 is required for recruitment of the core transcriptional machinery.

We hypothesize that *C. glabrata* THI promoters (with the exception of *CgPMU3*) behave similarly to *S. cerevisiae* promoters but only require Pdc2 and Thi3 (Figure 9B). This could be a consequence of the C-terminal activation domain as the two Pdc2 proteins are 80% identical in the N-terminal DNA binding domain half of the protein, but only 30% identical in the C-terminal region (Nosaka *et al.* 2012). This difference in Pdc2 proteins between the species could allow *Cg*Pdc2 to recruit RNA polymerase in a *THI2* independent manner and/or increase the affinity of the transcription factor for its DNA binding site.

*CgPMU3* presents a novel solution to thiamine starvation regulation. Whereas we can replace the *CgPMU3* UAS with the THI UAS and restore upregulation to some degree, the reverse is not true. This suggests that the *CgPMU3* UAS does not specifically recruit *Cg*Pdc2, but inclusion of the THI UAS now converts *CgPMU3* into a “standard” *C. glabrata* THI promoter. Additionally, introduction of a *Sc*Thi2 binding site into the *CgPMU3* promoter does not confer the same behavior as when it is introduced into the *CgTHI10* promoter. Thi2 in combination with Pdc2 confers upregulation in *CgPMU3*, but now the orientation of the site is irrelevant and the UAS is not required, suggesting that the only reason the *CgPMU3* promoter can work in *S. cerevisiae* is because of Thi2 recruitment to the promoter (Figure 8C). These data suggest two things. First, that the *CgPMU3* UAS is unlikely to bind Pdc2 with a high affinity, whereas the THI UAS likely does have a high affinity for Pdc2. Second, that Thi2 may be an important anchoring transcription factor in *S. cerevisiae* THI promoters as opposed to the Pdc2 transcription factor. This anchoring effect is reminiscent of Pho4 and Pho2 in *S. cerevisiae*, which regulate the induction of phosphate starvation genes. Pho4 has a well-defined recognition motif (GAGCTC), but Pho2 has a much more permissive recognition site (Zhou and O’Shea 2011; He *et al.* 2017). Our work suggests that there is a great deal of flexibility in the Pdc2 binding site and that Pdc2 may be binding to a relatively degenerate sequence. We believe that the *CgPMU3* promoter has acquired a novel mechanism for thiamine starvation regulation. It seems possible that a novel transcription factor has been co-opted into the THI pathway to act as a functional analog to Thi2, and it may bind both the *CgPMU3* UAS and *Cg*Pdc2 (Figure 9C). However, other possibilities exist, and we are investigating these possibilities.

Characterization of the *CgPMU3* promoter UAS has uncovered a potential new mechanism to regulate thiamine starvation genes and has demonstrated an interesting aspect of *cis* regulatory acquisition. Often, there is the recruitment of the same transcription factors, and thus, the apparent convergent evolution of the same *cis* sequences to bind those factors (Dalal *et al.* 2016; Cvekl *et al.* 2017; Kuang *et al.* 2018). However, we observe a novel *cis* regulatory sequence in a promoter that is recently evolved in a different genetic milieu (*i.e.*, lack of *THI2*), but still gives the same output as many other THI genes. It seems as if the simplest solution for this new promoter would be to evolve the standard ancestral THI UAS, but *CgPMU3* did not acquire that solution, either because of genetic constraints or because of genetic drift. However, the selective requirement for a thiamine repressible phosphatase important for the recycling of thiamine was likely present in the history of *C. glabrata* (Nahas *et al.* 2018). Therefore, it is possible that the lack of Thi2 in *C. glabrata* acted as a constraint in the evolution of the thiamine starvation induction of the *CgPMU3* gene, yielding the only high-fitness solution to the problem. Further dissection of how each promoter functions is required to understand the precise mechanism of *CgPMU3* upregulation. However, this work suggests that caution should be taken when investigating the incorporation of new genes into an existing regulatory pathway, as gain/loss of a constraint may change *cis* architecture in unforeseen ways.
